# Implications of the training of simulated skills and scenarios on the motivation for learning medical students: experimental study*

**DOI:** 10.1590/1518-8345.7661.4626

**Published:** 2025-10-27

**Authors:** Barbara Casarin Henrique-Sanches, Raphael Raniere de Oliveira Costa, Rodrigo Guimarães dos Santos Almeida, Rodrigo Magri Bernardes, Alessandra Mazzo

**Affiliations:** 1 Universidade de São Paulo, Escola de Enfermagem de Ribeirão Preto, PAHO/WHO Collaborating Centre for Nursing Research Development, Ribeirão Preto, SP, Brazil; 2 Scholarship holder at the Coordenação de Aperfeiçoamento de Pessoal de Nível Superior (CAPES), Brazil; 3 Universidade Federal do Rio Grande do Norte, Escola Multicampi de Ciências Médicas, Caicó, RN, Brazil; 4 Universidade Federal do Mato Grosso do Sul, Instituto Integrado de Saúde, Campo Grande, MS, Brazil; 5 Universidade de São Paulo, Faculdade de Medicina de Bauru, Bauru, SP, Brazil; 6 Scholarship holder at the Fundação de Amparo à Pesquisa do Estado de São Paulo (FAPESP), Brazil

**Keywords:** Health Human Resource Training, Education, Medical, Students, Medical, Simulation Training, Motivation, Learning

## Abstract

to verify the effect of skills training and simulated scenarios performed subsequently or belatedly on the motivation for learning of medical students.

experimental pre- and post-test study with control and intervention groups. Fifty second-year medical students participated and were randomized into two groups: control group (dialogical exposure, skills training and simulated scenarios after 12 hours) and intervention group (dialogical exposure, skills training and simulated scenarios after 21 days). The Situational Motivation Scale was used for analysis.

all students showed increased motivation. In the control group, intrinsic motivation increased before and after the simulated scenario (p=0.011). In the intervention group, intrinsic motivation increased before and after skills training (p=0.013), before and after the simulated scenario (p=0.024) and after skills training compared to the simulated scenario (p=0.011), with a reduction in amotivation (p=0.035).

skills training and simulated scenarios increase student motivation. However, there is a need to better understand the impacts of different intervals between activities.

## Highlights

**(1)** Simulated practices significantly increase students’ intrinsic motivation. **(2)** Larger time intervals between activities result in greater motivation. **(3)** Temporal optimization of activities can improve student engagement.

## Introduction

Education is a vast field that seeks to improve learning strategies, considering neurocognitive and behavioral aspects, such as cognition, emotions, and motivation^(1)^. In health education, clinical simulation stands out as an effective approach, allowing the integration and application of cognitive, psychomotor, and attitudinal knowledge^(2)^. However, its use requires careful planning, with clear objectives and appropriate materials^(3)^.

For simulation to be effective, activities must be organized progressively, allowing the structured development of students’ technical and behavioral skills^(4)^. Skills training, aimed at technical and attitudinal improvement, generally occurs through repetitive practice with anatomical models and other resources^(4)^. Simulated scenarios, which seek to reproduce complex clinical situations in a controlled environment, promote the development of clinical reasoning, decision-making, and teamwork^(4-5)^. To optimize learning, skills training often precedes simulated scenarios, respecting the progression of complexity^(5)^.

Motivation plays a central role in student engagement and learning and is influenced by both internal and external factors^(6)^. According to Self-Determination Theory, motivation can be intrinsic, driven by genuine interest and personal satisfaction, or extrinsic, guided by external rewards and social recognition^(6)^. Satisfaction of basic psychological needs—autonomy, competence, and belonging—strengthens motivation and facilitates the internalization of extrinsic motivation^(6)^.

Studies on motivation in simulation-based learning point to significant advantages, such as a positive environment that favors the development of technical and interpersonal skills, autonomy, a sense of belonging, and self-confidence^(2,7-8)^. However, there are still gaps in knowledge about how different simulated practice designs (skills training, simulated scenarios, and breaks between activities) influence motivation for learning.

Therefore, this study aims to verify the effect of skills training and simulated scenarios, carried out subsequently or belatedly, on medical students’ motivation for learning.

## Method

### Study design

This is an experimental pre-test and post-test study, following the CONSORT^(8)^ guidelines for simulation-based research. The study adopted a parallel design, with 1:1 allocation of participants into two distinct groups and blinding. Data collection was carried out between April and June 2022, and there were no methodological changes after its beginning^(9)^.

### Study setting

This study was conducted at the Skills and Simulation Laboratory of a public university in an inner city of the state of São Paulo, Brazil, with medical students. The course’s political-pedagogical project is based on active learning methods, including clinical simulation. The skills and simulation laboratory compose the structured physical space containing materials and equipment of various specificities. In addition, there are human resources who act as facilitators and professional.

### Population and sample

The eligible population consisted of second-year undergraduate medical students, and the sample was convenience-based.

The study included students regularly enrolled in the second year of the undergraduate course, who were over 18 years old and who had completed all activities and the data collection instruments. Students who were absent from any of the research activities and/or who completed the data collection instruments incompletely were excluded from the sample.

Fifty-six students participated in the first stage (lecture in a virtual learning environment). For stages 2 and 3, the participants were randomly allocated to two groups: Intervention Group (n=28) who underwent skills training followed by a 12-hour break and simulation scenarios; and Control Group (n=28) who underwent skills training followed by a 21-day break to perform the simulation scenarios.

**Figure 1-:**
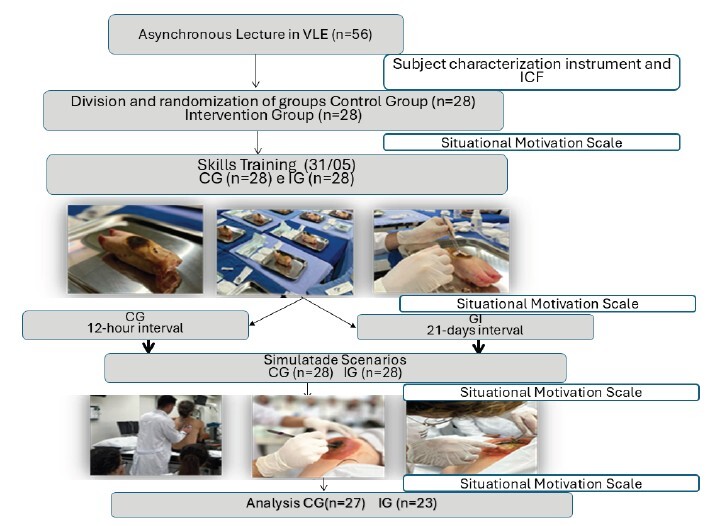
Flow diagram. Adapted from CONSORT^(8)^

After applying the inclusion criteria, the final sample consisted of 50 students. The process of selection, allocation and monitoring of the groups is presented in Figure 1

### Outcomes

The primary outcomes of this study predicted the existence of statistically significant differences in the levels of motivation for learning among medical students allocated to the different experimental groups, as well as significant variations in motivation scores between the different assessment moments within each group.

### Randomization

Participant allocation was performed by simple randomization, using the RAND() function of Microsoft Excel^®^. Each participant was registered with their university registration number, organized sequentially in an electronic spreadsheet, and the randomization sequence was applied. The allocation sequence was generated by an independent researcher, who had no contact with the participants during the intervention and later passed on to the facilitators responsible for implementing the experimental activities.

### Blinding

Due to the study design, it was not possible to blind the facilitators, as they needed to conduct the activities at specific times for each group. However, the participants were not aware of the temporal variable analyzed in the impact between the skills training and the simulated scenarios, which reduced the risk of expectation and response bias. In addition, the statistical analysis was conducted by a researcher blinded to the group allocation, minimizing bias in the interpretation of the results.

### Study development

The interventions took place during academic activities during the aging module and had as their main theme “Evaluation, prevention and treatment of chronic wounds”. The activity consisted of a lecture, skills training and simulation scenarios.

The theoretical basis of the activity was based on a bibliographic review and on the recommendations of the European Pressure Ulcer Advisory Panel, National Pressure Injury Advisory Panel and Pan Pacific Pressure Injury Alliance^(10)^, as well as on the guidelines of the International Nursing Association for Clinical Simulation and Learning^(4)^ and on Kolb’s Experiential Learning Theory^(11)^.

After planning and construction, the activities were validated in terms of face and content by experts and tested before their application. At this stage, no changes were suggested. The activities were developed by calibrated facilitators and divided into three stages.

The methodology adopted involved three main stages, the detailed configurations of which, including learning objectives, resources used and duration, are described in Figure 2.

Stage 1 – Asynchronous Lectures: Two asynchronous lectures were taught, each lasting two hours, made available in a virtual learning environment(VLE). The content covered skin anatomy, effects of skin aging, pathophysiology of chronic wounds and prevention and treatment strategies, including dressings, compression therapies and negative pressure therapy. In addition to the classes, students had access to complementary materials, such as instructional videos and clinical guidelines, available for 30 days in the virtual environment. During this period, the participants were also asked to fill out the questionnaire to characterize the participants.

Stage 2 – Skills Training: In the second stage, students participated in supervised practical training, focusing on risk assessment for injuries in diabetic patients with neuropathy and debridement of coagulation necrosis. The training followed a structured script, with practical demonstration, supervised execution and formative feedback at the end of the activity.

Stage 3 – Simulated Scenarios: Two simulated clinical scenarios were conducted, each lasting 90 minutes, including pre-briefing (10 min), simulation (20 min) and structured debriefing with formative feedback (60 min).

The cases addressed care situations in a basic health unit, as follows: Scenario 1 – Type 2 diabetic patient with numbness in the lower limbs, hyperkeratosis and fungal lesions, simulating a medical consultation in primary care.

Scenario 2 – Paraplegic patient with pressure injuries in different stages, requiring instrumental debridement of non-viable tissue. The scenario was carried out in a hybrid manner, using a professional actor and a low-fidelity simulator (pelvis attached to the simulated patient).

During the debriefings, students reflected on the experience, receiving structured feedback on clinical performance, safety in performing procedures, and communication aspects of the consultation.

Figure 2 below presents a breakdown of the teaching strategies, learning objectives, resources used, and intervention time at each stage of the study.

**Figure 2-:** Structure of teaching strategies, learning objectives, resources used and intervention time. Bauru, SP, Brazil, 2024

**Teaching and learning strategy**	**Learning objectives**	**Resources used**	**Intervention time**
Expository and asynchronous classes made available in a virtual learning environment	Recognize the anatomy of the skin; Understand the action of the aging process on skin tissue, as well as the etiology and pathophysiology of pressure injuries, vasculogenic injuries and diabetic neuropathy; Identify and classify viable and non-viable tissues and the staging of pressure injuries; Reflect on injury prevention strategies and injury management regarding the indication of coverings, correlates, elastic and inelastic compression therapies and negative pressure therapy.	Support videos: assessment of the foot of diabetic patients, measurement of the ankle-brachial index, application of the Unna boot, multilayer therapy, negative pressure therapy; Support materials: guidelines and scales for assessing the risk of developing pressure injuries.	4 hours
Simulated Scenario 1	Perform sensitivity assessment and screening tests on the feet of patients at risk of developing diabetic neuropathy. Perform instrumental debridement in coagulation necrosis and occlusive dressing	Stretcher; 128 Hz tuning fork; Semmes-Weinstein stensiometer with 10 g monofilament Fresh pig’s foot; Culinary blowtorch; Sterile glove; Scalpel blade; Scalpel handle; Anatomical forceps; Rat tooth forceps; Procedure tray; Sterile gauze; Simulated coverings and related materials; Microporous tape; Scissors; Polyurethane film; Waste bin for organic material; Waste bin for contaminated material; Rigid collection box for disposal of sharps.	4 hours
Simulated Scenario 1	Primary: Experience care in a basic health unit for a diabetic patient with impaired sensitivity in the feet with the presence of hyperkeratosis and fungal lesions. Secondary: Develop doctor-patient communication; Adopt biosafety principles; Develop clinical judgment skills; Provide care safely, based on scientific evidence; Implement prevention measures and guidance on self-care to prevent injuries.	128 Hz tuning fork; Semmes-Weinstein strain gauge with 10 g monofilament; *Moulage* ; Actor; Cotton; 70% alcohol.	90 minutes Pre-briefing: 10 min Scenario: 20 min Debriefing: 60 min
Simulated Scenario 2	Primary: Experience care in a basic health unit for a paraplegic patient with pressure injuries. Secondary: Develop doctor-patient communication; Adopt biosafety principles; Develop clinical judgment skills; Provide care safely, based on scientific evidence; Perform instrumental debridement of nonviable tissues; Apply dressings with coverings and related materials with the correct indication; Implement measures to prevent pressure injuries.	Wheelchair; Air cushion with central hole; Stretcher; Bedsheets; Low-fidelity simulator (pelvis); Molding compound; Artificial blood; Artificial burn; Banana peel; Red, brown, purple and yellow cream makeup; Scalpel blade; Scalpel handle; Procedure glove; Sterile glove; Anatomical forceps; Rat tooth forceps; Gauze; 70% alcohol; Simulated dressings and related materials; Scissors; Microporous tape.	90 minutes Pre-briefing: 10 min Scenario: 20 min Debriefing: 60 min.

### The intervention and the division of the Control Groups and the Experimental Group

After Stage 1, that is, after taking part in asynchronous classes in the virtual learning environment, students were randomized into a Control Group and an Experimental Group. From then on, the Control Group performed skills training (Stage 2) and after a 12-hour interval, participated in the simulated scenario (Stage 3) and the Intervention Group performed skills training (Stage 2) and, after a 21-day interval, participated in the simulated scenario (Stage 3). Before and after skills training, as well as before and after the simulated scenarios, both groups completed the Situational Motivation Scale^(12)^.

### Data collection instruments

Two data collection instruments were used. The first was a questionnaire with open and closed questions related to the academic and sociodemographic characteristics of the participants, including age, gender, financial support received from the university, and previous experience with the simulation theme. This instrument was applied after the development of the activities in the virtual learning environment.

The second instrument was the Situational Motivation Scale^(12)^, developed by Guay, Vallerand & Blanchard^(13)^ and translated and validated into Portuguese by Gamboa, Valadas & Paixão^(12)^. The Portuguese version demonstrated adequate construct and criterion validity, with confirmatory factor analysis supporting its four-factor structure, as recommended by Self-Determination Theory^(6)^. The reliability of the scale was measured by Cronbach’s alpha, ranging from 0.77 to 0.89, indicating good internal consistency^(12)^. The instrument is designed to assess situational motivation in an educational context and consists of 16 items, divided into 4 categories: Intrinsic Motivation (“Because I think this activity is interesting”), Identified Regulation (“Because it is for my own good”), External Regulation (“Because I can do it”) and Amotivation (“There may be good reasons for doing this activity, but I don’t see any”). Responses were recorded on a Likert scale, ranging from 1 (Does not correspond at all) to 7 (Exact correspondence).

## Ethical aspects

This study was approved by the Research Ethics Committee under number. 4,843,772, and all recommendations of Resolution 466 of 2012 of the National Health Council were followed^(14)^.

## Results

The average age of the participants was 21 years, and the majority were female. The majority also received financial support from the university, performed volunteer activities in the Skills and Simulation Laboratory, and had had previous contact with patients with chronic wounds. The sociodemographic and academic data characterizing the sample, by group, are shown in Table 1.

**Table 1–:** Sociodemographic profile of students. Bauru, SP, Brazil, 2024

**Characterization**		**Total**		**Intervention Group**		**Control Group**	
		Fr*	**%** ^†^	Fr*	**%** ^†^	Fr*	**%** ^†^
Gender	Female	35	70	16	70	19	70
	Male	15	30	7	30	8	30
Age	Minimum	18	-	18	-	18	-
	Maximum	32	-	32	-	32	-
	Average	21	-	21	-	21	-
	Median	21	-	21	-	21	-
Financial support from the university ^‡^	Receives	27	54	12	52	15	56
	Does not receive	23	46	11	48	12	44
Volunteer activities at LHS ^§^ in another shift	Performs	50	100	23	100	27	100
	Does not perform	0	0	0	0	0	0
Contact with patient with chronic wound	Yes	38	72	17	74	21	78
	No	12	28	6	26	6	22
Local contact with patient with wounds	UBS ^||^	19	38	8	35	11	41
	Family	7	14	3	13	4	15
	University Hospital	6	12	3	13	3	11
	Specialty Center	6	12	3	13	3	11

*Fr = Frequency; ^†^% = Percentage; ^‡^Financial aid granted to students to contribute to their permanence in higher education courses and graduation; ^§^LHS = Skills and Simulation Laboratory; ^||^UBS = Basic Health Unit

The Situational Motivation Scale^(12)^ showed high reliability in the sample studied (α=0.873) in all items. Table 2 presents descriptive statistics of the instrument values in relation to the motivation of the two groups of students, measured before and after the completion of the skills training and simulated scenarios.

**Table 2-:** Motivational profile for student learning before and after skills training and simulated scenario according to the Situational Motivation Scale^(12)^. Bauru, SP, Brazil, 2024

**Period**	**Scale domain**	**Control Group**				**Intervention Group**			
		Mean	Max*	Min ^†^	Standard deviation	Mean	Max*	Min ^†^	Standard deviation
Before Skills Training	Identified Regulation	6.2	7.0	4.3	0.765	6.3	7.0	4.3	0.724
	Intrinsic Motivation	5.4	7.0	3.0	1.215	5.6	7.0	4.0	0.881
	External Regulation	5.9	7.0	2.6	1.300	5.5	7.0	2.6	1.468
	Amotivation	1.5	3.2	1.0	0.702	1.2	2.2	1.0	0.336
After Skills Training	Identified Regulation	6.2	7.0	4.0	1.019	6.4	7.0	3.0	0.981
	Intrinsic Motivation	5.7	7.0	2.0	1.252	6.1	7.0	4.2	0.895
	External Regulation	5.8	7.0	2.3	1.409	5.5	7.0	2.0	1.689
	Amotivation	1.5	4.2	1.0	0.930	1.1	2.0	1.0	0.233
Before Simulated Scenario	Identified Regulation	6.2	7.0	4.3	0.860	6.2	7.0	4.6	0.729
	Intrinsic Motivation	5.4	7.0	2.5	1.352	5.4	7.0	4.0	0.922
	External Regulation	5.8	7.0	3.0	1.141	5.4	7.0	2.3	1.534
	Amotivation	1.3	4.0	1.0	0.651	1.3	3.5	1.0	0.607
After Simulated Scenario	Identified Regulation	6.4	7.0	4.0	0.902	6.3	7.0	5.0	0.788
	Intrinsic Motivation	5.7	7.0	2.7	1.191	5.7	7.0	4.2	0.903
	External Regulation	5.9	7.0	3.3	1.197	5.4	7.0	1.3	1.598
	Amotivation	1.3	3.5	1.0	0.584	1.3	2.7	1.0	0.505

*Max = Maximum; ^†^Min = Minimum

The sample presented an abnormal distribution (Kolmogorov-Smirnov p >.001); therefore, to compare the students’ motivation before and after the activities within each group, the Wilcoxon Test was used. Among the individuals in the same group, the data that showed significant differences in the Control Group were Intrinsic Motivation before versus after the simulated scenario, and in the Intervention Group differences in Intrinsic Motivation before versus after the skills training, before versus after the simulated scenario and after the skills training versus after the simulated scenario, with the last moment also being significant in the Amotivation of the Intervention Group. The data are presented in Table 3.

**Table 3-:** Comparison between the group’s own domains of the Situational Motivation Scale^(12)^ in carrying out the proposed activities at the four different times. Bauru, SP, Brazil, 2024

**Moment**	**Compared Domains**	ρ* CG ^†^	ρ* IG ^‡^
Before and after skill training	Intrinsic Motivation vs ^§^ Intrinsic Motivation	-	0.013
Before and after simulated scenario	Intrinsic Motivation vs ^§^ Intrinsic Motivation	0.011	0.024
After skill training and simulated scenario	Intrinsic Motivation vs ^§^ Intrinsic Motivation	-	0.011
After skill training and simulated scenario	Amotivation vs ^§^ Amotivation	-	0.035

*ρ = Significance Level Wilcoxon Test; ^†^CG = Control Group; ^‡^IG = Intervention Group; ^§^vs = Versus

The domains of the Situational Motivation Scale^(12)^ were also compared between the Control Group and the Intervention Group at all times evaluated. The results show that there were no significant differences in student motivation in any of the periods analyzed (Mann-Whitney p ≥ 0.05) in student motivation.

## Discussion

As shown in the results, most participants received some type of financial aid. In Brazil, student retention policies were implemented to mitigate inequalities, aiming to democratize access to higher education. However, student retention at university can be challenging due to the demands and conditions of courses, such as medicine, which require exclusive dedication, which can result in psychological distress and dropout. To face these challenges and strengthen the sense of belonging, Brazilian universities offer socioeconomic aid, such as student housing, university restaurants, and financial aid^(15)^. The sense of belonging plays a relevant role in the student’s academic success and emotional well-being, reflecting their connection and identification with the learning environment. When students feel they belong as an integral part of this environment, they demonstrate greater engagement in the knowledge acquisition process^(7)^.

It was observed that most participants carried out volunteer activities in the Skills and Simulation Laboratory. The main objective of these types of laboratories is to immerse students in the culture and social aspects of the clinical environment, contributing to the formation of professional values essential for clinical practice. This experience seeks to promote safety, confidence and reduce anxiety, allowing students to try new approaches and accept mistakes as an integral part of their learning process. In addition, participation in these activities is associated with the development of autonomy, competence and collaboration among peers^(2)^.

Several elements influence student motivation, including the style and motivation of teachers, teaching activities, curricular organization, academic context and early insertion into practice environments, as recommended by the National Curricular Guidelines for medical courses^(16)^.

To stimulate student motivation in higher education, teachers must act as facilitators, adopting dynamic and creative methods that arouse student interest. The use of active teaching methods promotes greater motivation for learning, since motivated students seek to understand and find meaning in the knowledge acquired. When addressing student motivation, the activation of stimulus mechanisms can intervene in the teaching and learning dynamics, leading to notable impacts on the knowledge acquisition process, as well as on autonomy, knowledge and self-confidence^(7)^. A study that used active methods demonstrated increased intrinsic motivation values and decreased external regulation^(17)^.

Addressing numerous situations in which the student experiences the applicability of their professional practice, which can lead them to internalize the need to learn to be a good professional, the use of simulation-based teaching is a powerful tool through the application of skills training and simulated scenarios^(5)^. In this study, the repercussions of skills training and simulated scenarios were beneficial for motivation, increasing the average motivational scores after carrying out the activities of the two groups regarding Identified Regulation and Intrinsic Motivation, and decreasing External Regulation and Amotivation (Table 3 Table 2), as already found in previous studies^(18)^.

Intrinsic Motivation is related to the innate pleasure in performing the activity, without interest in external gains^(6)^. Identified Regulation indicates that students have internalized the reasons why the activity was being performed and, even if there is no innate interest, they recognize its importance for professional practice^(6)^.

Autonomous motivation, which encompasses the previous items, is driven by internal factors and is aligned with the student’s individual needs, values, and interests. Although there may be an external source of motivation, such as a reward or incentive, when autonomous motivation predominates, the student internalizes the reasons for the activity and recognizes its relevance to their personal goals, values, or identity^(6)^.

Autonomous motivation is considered the most desirable type of motivation to be awakened in students, as it is associated with better learning outcomes, higher performance, greater commitment and well-being^(6)^. In the context of medical education, students with autonomous motivation are more likely to present proactive learning behaviors, such as seeking challenging clinical experiences and engaging in reflective practices^(17)^.

The significant increase in intrinsic motivation observed after participation in simulated clinical scenarios may be related to the meaning of autonomy provided by these activities. Simulated clinical scenarios allow students to associate the skills learned in training with the practice of competencies, increasing feelings of self-confidence, self-efficacy and satisfaction, especially when accompanied by adequate feedback and emotional support^(18)^.

Regarding the increase in Intrinsic Motivation in relation to simulated scenarios compared to skills training (p=0.011) and the decrease in Amotivation (p=0.035) perceived in the intervention group of this sample, it is related to studies that indicate that longer intervals between teaching activities favor consolidation processes, such as mental repetitions, integration with previous knowledge, strengthening of neural connections, increased familiarity and fluency with the topic, self-assessment and autonomous review^(19)^. These factors may have implications for competence, self-efficacy, belonging, satisfaction and autonomy for the development of the proposed activity. These findings are in line with studies carried out with elementary and high school students and with undergraduate students in biological sciences, which compared the motivation for learning in students submitted to activities with instructional design with the application of spaced activities, resulting in better motivational levels for learning^(20-22)^. However, in this sample, it was not possible to confirm the hypothesis that the spacing between skills training and simulated scenarios would significantly increase student motivation (Mann-Whitney test > 0.05). There are numerous studies that point to the benefits of time intervals in simulated activities, compared to those performed subsequently. However, these studies evaluate the effects on the retention of skills, abilities and knowledge, without specific judgments regarding their effects on motivation for learning^(5,23)^.

Motivation plays a crucial role in the performance of medical students during skills training and simulated scenarios. Motivated students not only engage more in simulation activities but also demonstrate improved knowledge and development of attitudes essential for clinical practice. Improving health education, especially through simulation, strengthens the preparation of future physicians, allowing them to reach internships more confident and capable. The potential of a motivated student extends beyond training, positively influencing the quality of care provided and patient safety throughout their professional career. Therefore, it is essential to create learning environments that encourage motivation and maximize the benefits of simulation, ensuring more effective medical training and, consequently, safer and more qualified patient care^(24)^.

As for the limitations of the study, the sample consisted of students from a single institution, which may limit the generalization of the results to other academic populations. Furthermore, individual factors that influence motivation, such as personal characteristics and previous experiences, were not explored in depth.

Despite the limitations, the findings contribute to the improvement of simulation-based teaching, offering empirical support for pedagogical planning in health courses. The identification of motivational impacts associated with skills training and simulated scenarios reinforces the importance of structuring activities in a progressive and strategically spaced manner.

## Conclusion

The results demonstrated an increase in Intrinsic Motivation (p=0.011) and a decrease in Amotivation (p=0.035) among students who performed the skills training and simulated scenarios with a longer time interval between their executions. These results are relevant for planning academic activities, so that they are motivating and lead to new purposes that can more accurately explore the motivational impacts on medical students. The present study can also foster new approaches to the potential of simulation in motivating students and the various outcomes after the simulated experience in training, as well as throughout the professional life of those who enjoyed the teaching method.

## Data Availability

All data generated or analysed during this study are included in this published article.
