# Supplementary material for: Implications of the training of simulated skills and scenarios on the motivation for learning medical students: experimental study
Source: Rev Lat Am Enfermagem. 2025 Oct 27;33:e4626. doi: 10.1590/1518-8345.7661.4626 (PMC12562552; doi:10.1590/1518-8345.7661.4626)
Supplement: Translation

# Translation: Implicações do treino de habilidades e cenários simulados na motivação para a aprendizagem de estudantes de medicina: estudo experimental*

**DOI:** 10.1590/1518-8345.7661.4627

**Authors:** Barbara Casarin Henrique-Sanches, Raphael Raniere de Oliveira Costa, Rodrigo Guimarães dos Santos Almeida, Rodrigo Magri Bernardes, Alessandra Mazzo

**Affiliations:** 1 Universidade de São Paulo, Escola de Enfermagem de Ribeirão Preto, Centro Colaborador da OPAS/OMS para o Desenvolvimento da Pesquisa em Enfermagem, Ribeirão Preto, SP, Brasil; 2 Bolsista da Coordenação de Aperfeiçoamento de Pessoal de Nível Superior (CAPES), Brasil; 3 Universidade Federal do Rio Grande do Norte, Escola Multicampi de Ciências Médicas, Caicó, RN, Brasil; 4 Universidade Federal do Mato Grosso do Sul, Instituto Integrado de Saúde, Campo Grande, MS, Brasil; 5 Universidade de São Paulo, Faculdade de Medicina de Bauru, Bauru, SP, Brasil; 6 Bolsista da Fundação de Amparo à Pesquisa do Estado de São Paulo (FAPESP), Brasil

**Keywords:** Capacitação de Recursos Humanos em Saúde, Educação Médica, Estudantes de Medicina, Treinamento por Simulação, Motivação, Aprendizagem

## Abstract

verificar o efeito do treino de habilidades e dos cenários simulados realizados de forma subsequente ou tardia na motivação para aprendizagem de estudantes de medicina.

estudo experimental pré e pós-teste com grupo controle e intervenção. Participaram 50 estudantes do segundo ano de medicina, randomizados em dois grupos: grupo controle (exposição dialogada, treino de habilidades e cenários simulados após 12h) e grupo intervenção (exposição dialogada, treino de habilidades e cenários simulados após 21 dias). A Escala de Motivação Situacional foi utilizada para análise.

todos os estudantes apresentaram aumento na motivação. No grupo controle, a motivação intrínseca aumentou antes e após o cenário simulado (p=0,011). No grupo intervenção, a motivação intrínseca aumentou antes e após o treino de habilidades (p=0,013), antes e após o cenário simulado (p=0,024) e após o treino de habilidades comparado ao cenário simulado (p=0,011), com redução da amotivação (p=0,035).

o treino de habilidades e os cenários simulados aumentam a motivação dos estudantes. Contudo, é necessário compreender melhor os impactos dos diferentes intervalos entre as atividades.

## Destaques

**(1)** Práticas simuladas aumentam significativamente a motivação intrínseca dos estudantes. **(2)** Intervalos temporais maiores entre atividades resultam em maior motivação. **(3)** Otimização temporal de atividades pode melhorar o engajamento dos estudantes.

## Introdução

A educação é um campo vasto que busca aprimorar estratégias de aprendizagem, considerando aspectos neurocognitivos e comportamentais, como cognição, emoções e motivação ^( 1 )^. No ensino em saúde, a simulação clínica se destaca como uma abordagem eficaz, permitindo a integração e aplicação de conhecimentos cognitivos, psicomotores e atitudinais ^( 2 )^. Contudo, seu uso exige um planejamento cuidadoso, com objetivos claros e materiais apropriados ^( 3 )^.

Para que a simulação seja eficaz, as atividades devem ser organizadas de forma progressiva, permitindo o desenvolvimento estruturado das competências técnicas e comportamentais dos estudantes ^( 4 )^. O treino de habilidades, voltado para o aprimoramento técnico e atitudinal, geralmente ocorre por meio da prática repetitiva com modelos anatômicos e outros recursos ^( 4 )^. Já os cenários simulados, que buscam reproduzir situações clínicas complexas em um ambiente controlado, promovem o desenvolvimento do raciocínio clínico, tomada de decisão e trabalho em equipe ^( 4 - 5 )^. Para otimizar a aprendizagem, os treinos de habilidades frequentemente precedem os cenários simulados, respeitando a progressão de complexidade ^( 5 )^.

A motivação desempenha um papel central no engajamento e na aprendizagem dos estudantes, sendo influenciada por fatores internos e externos ^( 6 )^. Segundo a Teoria da Autodeterminação, a motivação pode ser intrínseca, impulsionada pelo interesse genuíno e pela satisfação pessoal, ou extrínseca, orientada por recompensas externas e reconhecimento social ^( 6 )^. A satisfação das necessidades psicológicas básicas — autonomia, competência e pertencimento — fortalece a motivação e facilita a internalização da motivação extrínseca ^( 6 )^.

Estudos sobre a motivação no ensino baseado em simulação apontam vantagens significativas, como um ambiente positivo que favorece o desenvolvimento de habilidades técnicas, interpessoais, autonomia, senso de pertencimento e autoconfiança ^( 2 , 7 - 8 )^. No entanto, ainda há lacunas no conhecimento sobre como diferentes designs de prática simulada (treino de habilidades, cenários simulados e intervalos entre atividades) influenciam a motivação para a aprendizagem.

Dessa forma, este estudo tem como objetivo verificar o efeito do treino de habilidades e dos cenários simulados, realizados de forma subsequente ou tardia, na motivação para a aprendizagem de estudantes de medicina.

## Método

### Desenho do estudo

Trata-se de um estudo experimental do tipo pré-teste e pós-teste, seguindo as diretrizes do CONSORT ^( 8 )^ para pesquisas baseadas em simulação. O estudo adotou um design paralelo, com alocação 1:1 dos participantes em dois grupos distintos e com cegamento. A coleta de dados foi realizada entre os meses de abril e junho de 2022, e não houve alterações metodológicas após seu início ^( 9 )^.

### Local do estudo

Este estudo foi realizado no Laboratório de Habilidades e Simulação de uma universidade pública do interior do estado de São Paulo, Brasil, com estudantes de um curso de medicina. O referido curso tem o seu projeto político-pedagógico baseado em métodos ativos de aprendizagem, incluindo a simulação clínica. O laboratório de habilidades e simulação é um espaço físico e estruturado contendo materiais e equipamentos, de diversas especificidades. Ademais, conta com recursos humanos que atuam como facilitadores e atores profissionais.

### População e amostra

A população elegível foi composta por estudantes do segundo ano de graduação em medicina, e a amostra foi por conveniência.

Foram incluídos no estudo os estudantes regularmente matriculados no segundo ano do curso de graduação, maiores de 18 anos, que cumpriram todas as atividades e que preencheram integralmente os instrumentos de coleta de dados. Foram excluídos da amostra os estudantes que se ausentaram de qualquer uma das atividades da pesquisa e ou que realizaram o preenchimento incompleto dos instrumentos de coleta de dados.

Cinquenta e seis estudantes participaram da primeira etapa (aula expositiva em ambiente virtual de aprendizagem). Para as etapas 2 e 3, os participantes foram alocados aleatoriamente em dois grupos: Grupo Intervenção (n=28), os quais realizaram treino de habilidades seguido por intervalo de 12 horas e realização de cenários simulados; e Grupo Controle (n=28), que realizaram treino de habilidades seguido de intervalo de 21 dias para realização dos cenários simulados.

Após a aplicação dos critérios de inclusão, a amostra final foi constituída por 50 estudantes. O processo de seleção, alocação e seguimento dos grupos está apresentado na Figura 1.

**Figura 1-:** Diagrama de fluxo. Adaptado do CONSORT ^( 8 )^

### Desfechos

Os desfechos primários deste estudo previam a existência de diferenças estatisticamente significativas nos níveis de motivação para a aprendizagem entre os estudantes de medicina alocados nos diferentes grupos experimentais, bem como variações significativas nos escores de motivação entre os diferentes momentos de avaliação dentro de cada grupo.

### Randomização

A alocação dos participantes foi realizada por meio de randomização simples, utilizando a função RAND() do *Microsoft Excel* ^®^. Cada participante foi cadastrado conforme o número de registro universitário, organizado sequencialmente em planilha eletrônica, de modo que a sequência de randomização foi aplicada. A sequência de alocação foi gerada por um pesquisador independente, que não teve contato com os participantes durante a intervenção e, posteriormente, repassada aos facilitadores responsáveis pela aplicação das atividades experimentais.

### Cegamento

Devido ao desenho do estudo, não foi possível cegar os facilitadores, pois eles precisavam conduzir as atividades nos momentos específicos de cada grupo. No entanto, os participantes não tinham conhecimento da variável temporal analisada no impacto entre o treino de habilidades e os cenários simulados, o que reduziu o risco de viés de expectativa e resposta. Além disso, a análise estatística foi conduzida por um pesquisador cego para a alocação dos grupos, minimizando possíveis vieses na interpretação dos resultados.

### Desenvolvimento do estudo

As intervenções ocorreram durante as atividades acadêmicas no decorrer do módulo de envelhecimento e tiveram como tema principal “Avaliação, prevenção e tratamento de feridas crônicas”. A atividade foi composta por aula expositiva, treino de habilidades e cenários de simulação.

A fundamentação teórica da atividade foi baseada em revisão bibliográfica e nas recomendações do guia *European Pressure Ulcer Advisory Panel, National Pressure Injury Advisory Panel* e da *Pan Pacific Pressure Injury Alliance* ^( 10 )^, bem como nas diretrizes da *International Nursing Association for Clinical Simulation and Learning* ^( 4 )^ e na Teoria da Aprendizagem Experiencial de Kolb ^( 11 )^.

Após o planejamento e construção, as atividades foram validadas por especialistas quanto à face e ao conteúdo e testadas antes de sua aplicação. Nessa primeira etapa, não foram sugeridas alterações. As atividades foram desenvolvidas por facilitadores calibrados e divididas em três momentos principais, cujas configurações detalhadas, incluindo os objetivos de aprendizagem, os recursos utilizados e o tempo de duração, estão descritas na Figura 2.

Etapa 1 – Aulas Expositivas Assíncronas: ministraram-se duas aulas expositivas assíncronas, cada uma com duração de duas horas, disponibilizadas em um ambiente virtual de aprendizagem. Os conteúdos abordaram a anatomia da pele, efeitos do envelhecimento cutâneo, fisiopatologia de feridas crônicas e estratégias de prevenção e tratamento, incluindo coberturas, terapias compressivas e terapia por pressão negativa. Além das aulas, os estudantes tiveram acesso a materiais complementares, como vídeos instrucionais e *guidelines* clínicos, disponíveis por 30 dias no ambiente virtual. Neste período, também foi realizado o preenchimento do questionário de caracterização dos participantes.

Etapa 2 – Treino de Habilidades: na segunda etapa, os estudantes participaram de um treinamento prático supervisionado, com foco na avaliação de risco para lesões em pacientes diabéticos com neuropatia e no desbridamento de necrose de coagulação. O treinamento seguiu um roteiro estruturado, com demonstração prática, execução supervisionada e *feedback* formativo ao final da atividade.

Etapa 3 – Cenários Simulados: foram conduzidos dois cenários clínicos simulados, cada um com 90 minutos de duração, incluindo pré-*briefing* (10 min), simulação (20 min) e *debriefing* estruturado com *feedback* formativo (60 min).

Os casos abordaram situações de atendimento em unidade básica de saúde, sendo: Cenário 1 – Paciente diabético tipo 2 com insensibilidade nos membros inferiores, hiperqueratose e lesões fúngicas, simulando uma consulta médica em atenção primária.

Cenário 2 – Paciente paraplégico com lesões por pressão em diferentes estágios, necessitando desbridamento instrumental de tecidos inviáveis. O cenário foi realizado de forma híbrida, utilizando um ator profissional e simulador de baixa fidelidade (pelve acoplada ao paciente simulado).

Durante os *debriefings*, os estudantes refletiram sobre a experiência, recebendo *feedback* estruturado sobre o desempenho clínico, a segurança na execução dos procedimentos e os aspectos comunicacionais da consulta.

A Figura 2, a seguir, apresenta um detalhamento das estratégias de ensino, objetivos de aprendizagem, recursos utilizados e tempo de intervenção em cada etapa do estudo.

**Figura 2-:** Estrutura das estratégias de ensino, objetivos de aprendizagem, recursos utilizados e tempo de intervenção. Bauru, SP, Brasil, 2024

**Estratégia de ensino e aprendizagem**	**Objetivos de aprendizagem**	**Recursos utilizados**	**Tempo de intervenção**
Aulas expositivas e assíncronas disponibilizadas em ambiente virtual de aprendizagem	Reconhecer a anatomia da pele; Compreender ação do processo de envelhecimento no tecido cutâneo, bem como a etiologia e fisiopatologia de lesões por pressão, vasculogênicas e por neuropatia diabética; Identificar e classificar tecidos viáveis, inviáveis e estadiamento das lesões por pressão; Refletir acerca das estratégias de prevenção de lesões e o manejo da lesão quanto indicação de coberturas, correlatos, terapias compressivas elásticas, inelásticas e terapia por pressão negativa.	Vídeos de apoio: avaliação do pé do paciente diabético, realização da mensuração do índice tornozelo braço, aplicação de bota de *unna*, terapia multicamadas, terapia por pressão negativa; Materiais de apoio: *guidelines* e escalas de avaliação de risco de desenvolvimento de lesão por pressão.	4 horas
Treino de habilidades	Realizar teste de avaliação de sensibilidade e rastreamento em pés de pacientes com risco de desenvolver neuropatia diabética. Realizar desbridamento instrumental em necrose de coagulação e curativo oclusivo	Maca; Diapasão de 128 Hz; Estensiômetro Semmes-Weinstein com monofilamento de 10 g Pé de porco *in natura;* Maçarico culinário; Luva estéril; Lâmina de bisturi; Cabo de bisturi; Pinça anatômica; Pinça dente de rato; Bandeja para procedimento; Gaze estéril; Coberturas e correlatos simulados; Fita microporosa; Tesoura; Filme de poliuretano; Lixeira para material orgânico; Lixeira para material contaminado; Caixa coletora rígida para descarte de perfurocortante.	4 horas
Cenário Simulado 1	Primários: Vivenciar um atendimento em unidade básica de saúde com paciente diabético com sensibilidade nos pés prejudicadas com presença de hiperqueratose e lesões fúngicas. Secundários: Desenvolver a comunicação médico-paciente; Adotar os princípios de biossegurança; Desenvolver a competência de julgamento clínico; Prestar assistência de forma segura, baseada em evidências científicas; Implementar medidas de prevenção e orientação sobre autocuidado ao para prevenção de lesões.	Diapasão de 128 Hz; Entensiometro Semmes-Weinstein com monofilamento de 10 g; *Moulage* ; Ator; Algodão; Álcool 70%.	90 minutos Pré *Briefing*: 10 min Cenário: 20 min *Debriefing*: 60 min
Cenário Simulado 2	Primário: Vivenciar um atendimento em unidade básica de saúde com paciente paraplégico com lesões por pressão. Secundários: Desenvolver a comunicação médico-paciente; Adotar os princípios de biossegurança; Desenvolver a competência de julgamento clínico; Prestar assistência de forma segura, baseada em evidências científicas; Realizar desbridamento instrumental de tecidos inviáveis; Realizar curativo com coberturas e correlatos com indicação correta; Implementar medidas de prevenção de lesões por pressão.	Cadeira de rodas; Almofada de ar com orifício central; Maca; Lençóis; Simulador de baixa fidelidade (pelve); Massa de moldagem; Sangue artificial; Queimadura artificial; Casca de banana; Maquiagem em creme vermelha, marrom, roxa e amarela; Lâmina de bisturi; Cabo de bisturi; Luva de procedimento; Luva estéril; Pinça anatômica; Pinça dente de rato; Gaze; Álcool 70%; Coberturas e correlatos simulados; Tesoura; Fita microporosa.	90 minutos Pré *Briefing*: 10 min Cenário: 20 min *Debriefing*: 60 min.

### A intervenção e a divisão dos Grupos Controle e do Grupo Experimental

Após a Etapa 1, ou seja, após assistirem às aulas assíncronas no ambiente virtual de aprendizagem, os estudantes foram randomizados em Grupo Controle e em Grupo Experimental. A partir de então, o Grupo Controle realizou o treino de habilidades (Etapa 2) e após um intervalo de 12 horas, participou do cenário simulado (Etapa 3). Já o Grupo Intervenção realizou o treino de habilidades (Etapa 2) e, após um intervalo de 21 dias, participou do cenário simulado (Etapa 3). Antes e após o treino de habilidades, bem como antes e após os cenários simulados, ambos os grupos preencheram a Escala de Motivação Situacional ^( 12 )^.

### Instrumentos de coleta de dados

Foram utilizados dois instrumentos de coleta de dados. O primeiro foi um questionário com perguntas abertas e fechadas, relacionadas à caracterização acadêmica e sociodemográficas dos participantes, incluindo idade, gênero, apoios financeiros recebidos da universidade, experiência prévia com o tema da simulação. Este instrumento foi aplicado após o desenvolvimento das atividades no ambiente virtual de aprendizagem.

O segundo instrumento foi a Escala de Motivação Situacional ^( 12 )^, desenvolvida por Guay, Vallerand & Blanchard ^( 13 )^ e traduzido e validado para o português por Gamboa, Valadas & Paixão ^( 12 )^. A versão em português demonstrou validade de constructo e critério adequadas, com análise fatorial confirmatória, sustentando sua estrutura em quatro fatores, conforme preconizado pela Teoria da Autodeterminação ^( 6 )^. A confiabilidade da escala foi aferida pelo alfa de Cronbach, variando entre 0.77 e 0.89, indicando boa consistência interna ^( 12 )^.

O instrumento destina-se a avaliar a motivação situacional num contexto educacional e é composto por 16 itens, divididos em 4 categorias: Motivação Intrínseca (“Porque penso que esta atividade é interessante”), Regulação Identificada (“Porque é para o meu próprio bem”), Regulação Externa (“Porque o posso fazer”) e Amotivação (“Pode haver boas razões para fazer esta atividade, mas não vejo nenhuma”). As respostas foram registradas em uma escala Likert, variando de 1 (Não corresponde de todo) a 7 (Correspondência exata).

## Aspectos éticos

Este estudo foi aprovado pelo Comitê de Ética em Pesquisa sob o parecer nº 4.843.772, e todas as recomendações da Resolução 466 de 2012 do Conselho Nacional de Saúde foram seguidas ^( 14 )^.

## Resultados

A idade média dos participantes foi de 21 anos, sendo a maioria do sexo feminino. A maioria também recebia apoio financeiro da universidade, realizava atividades voluntárias no Laboratório de Habilidades e Simulação, e já havia tido contato prévio com pacientes com ferida crônica. Os dados sociodemográficos e acadêmicos de caracterização da amostra, por grupo, estão dispostos na Tabela 1.

**Tabela 1-:** Perfil sociodemográfico dos estudantes. Bauru, SP, Brasil, 2024

**Caracterização**		**Total**		**Grupo Intervenção**		**Grupo Controle**	
		Fr*	**%** ^†^	Fr*	**%** ^†^	Fr*	**%** ^†^
Sexo	Feminino	35	70	16	70	19	70
	Masculino	15	30	7	30	8	30
Idade	Mínimo	18	-	18	-	18	-
	Máximo	32	-	32	-	32	-
	Média	21	-	21	-	21	-
	Mediana	21	-	21	-	21	-
Apoio financeiro da universidade ^‡^	Recebe	27	54	12	52	15	56
	Não recebe	23	46	11	48	12	44
Atividades voluntárias no LHS ^§^ em outro turno	Realiza	50	100	23	100	27	100
	Não Realiza	0	0	0	0	0	0
Contato com paciente com ferida crônica	Sim	38	72	17	74	21	78
	Não	12	28	6	26	6	22
Local contato com paciente com feridas	UBS ^||^	19	38	8	35	11	41
	Familiares	7	14	3	13	4	15
	Hospital Universitário	6	12	3	13	3	11
	Centro de Especialidade	6	12	3	13	3	11

*Fr = Frequência; ^†^% = Porcentagem; ^‡^Auxílio financeiro concedido aos estudantes para contribuir em sua permanência nos cursos superiores e diplomação; ^§^LHS = Laboratório de Habilidades e Simulação; ^||^UBS = Unidade Básica de Saúde

A Escala de Motivação Situacional ^( 12 )^ apresentou alta confiabilidade na amostra estudada (α=0,873) em todos os itens. A Tabela 2 apresenta estatística descritiva dos valores do instrumento em relação à motivação dos dois grupos de estudantes, mensurada antes e após a realização do treino de habilidades e dos cenários simulados.

**Tabela 2-:** Perfil motivacional para a aprendizagem dos estudantes antes e após as treino de habilidades e cenário simulado segundo Escala de Motivação Situacional^(12)^. Bauru, SP, Brasil, 2024

**Período**	**Domínio Escala**	**Grupo Controle**				**Grupo Intervenção**			
		Média	Máx*	Min ^†^	Desvio-padrão	Média	Máx*	Min ^†^	Desvio-padrão
Antes Treino de Habilidades	Regulação Identificada	6,2	7,0	4,3	0,765	6,3	7,0	4,3	0,724
	Motivação Intrínseca	5,4	7,0	3,0	1,215	5,6	7,0	4.0	0,881
	Regulação Externa	5,9	7,0	2,6	1,300	5,5	7,0	2,6	1,468
	Amotivação	1,5	3,2	1,0	0,702	1,2	2,2	1,0	0,336
Após Treino de Habilidades	Regulação Identificada	6,2	7,0	4,0	1,019	6,4	7,0	3,0	0,981
	Motivação Intrínseca	5,7	7,0	2,0	1,252	6,1	7,0	4,2	0,895
	Regulação Externa	5,8	7,0	2,3	1,409	5,5	7,0	2,0	1,689
	Amotivação	1,5	4,2	1,0	0,930	1,1	2,0	1,0	0,233
Antes Cenário Simulado	Regulação Identificada	6,2	7,0	4,3	0,860	6,2	7,0	4,6	0,729
	Motivação Intrínseca	5,4	7,0	2,5	1,352	5,4	7,0	4,0	0,922
	Regulação Externa	5,8	7,0	3,0	1,141	5,4	7,0	2,3	1,534
	Amotivação	1,3	4,0	1,0	0,651	1,3	3,5	1,0	0,607
Após Cenário Simulado	Regulação Identificada	6,4	7,0	4,0	0,902	6,3	7,0	5,0	0,788
	Motivação Intrínseca	5,7	7,0	2,7	1,191	5,7	7,0	4,2	0,903
	Regulação Externa	5,9	7,0	3.3	1,197	5,4	7,0	1,3	1,598
	Amotivação	1,3	3,5	1,0	0,584	1,3	2,7	1,0	0,505

*Max = Máximo; ^†^Min = Mínimo

A amostra apresentou distribuição anormal (Kolmogorov-Smirnov p >,001), portanto, para comparar a motivação dos estudantes antes e após as atividades dentro de cada grupo, utilizou-se o Teste de Wilcoxon*.* Entre os indivíduos do mesmo grupo, os dados que evidenciaram diferenças significativas no Grupo Controle foram: Motivação Intrínseca antes *versus* pós o cenário simulado, e, no Grupo Intervenção, as diferenças se deram na Motivação Intrínseca antes *versus* após o treino de habilidades, antes *versus* após o cenário simulado e após o treino de habilidades *versus* após cenário simulado, sendo o último momento também significativo na Amotivação do Grupo Intervenção. Os dados estão apresentados na Tabela 3.

**Tabela 3-:** Comparação entre o próprio grupo dos domínios da Escala de Motivação Situacional ^( 12 )^ na realização das atividades propostas nos quatro momentos distintos. Bauru, SP, Brasil, 2024

**Momento**	**Domínios Comparados**	ρ* GC ^†^	ρ* **GI** ^‡^
Antes e após treino de habilidade	Motivação Intrínseca *vs* ^§^ Motivação Intrínseca	-	0,013
Antes e após cenário simulado	Motivação Intrínseca *vs* ^§^ Motivação Intrínseca	0,011	0,024
Após treino de habilidade e cenário simulado	Motivação Intrínseca *vs* ^§^ Motivação Intrínseca	-	0,011
Após treino de habilidade e cenário simulado	Amotivação *vs* ^§^ Amotivação	-	0,035

*ρ = Nível de Significância Teste de Wilcoxon; ^†^GC = Grupo Controle; ^‡^GI = Grupo Intervenção; ^§^ *vs* = *Versus*

Também foi realizada a comparação dos domínios da Escala de Motivação Situacional ^( 12 )^ entre os Grupos Controle e Grupo Intervenção em todos os momentos avaliados. Os resultados demonstram que não houve diferenças significativas na motivação dos estudantes em nenhum dos períodos analisados (Mann-Whitney p ≥ 0,05).

## Discussão

Conforme apresentado nos resultados, a maioria dos participantes recebia algum tipo de auxílio financeiro. No Brasil, as políticas de permanência estudantil foram implementadas para mitigar desigualdades, visando democratizar o acesso ao ensino superior. No entanto, a permanência do estudante na universidade pode ser desafiadora devido às exigências e condições dos cursos, como os de medicina, que requerem dedicação exclusiva, o que pode resultar em sofrimento psicológico e evasão. Para enfrentar esses desafios e fortalecer o sentimento de pertencimento, as universidades brasileiras oferecem auxílios socioeconômicos, como moradia estudantil, restaurante universitário e auxílio financeiro ^( 15 )^. O sentimento de pertencimento desempenha um papel relevante no seu sucesso acadêmico e bem-estar emocional do estudante, refletindo sua conexão e identificação com o ambiente de aprendizagem. Quando os estudantes se sentem parte integrante desse ambiente, demostram maior engajamento no processo de aquisição de conhecimento ^( 7 )^.

Observou-se que a maioria dos participantes realizava atividades voluntárias no Laboratório de Habilidades e Simulação. Esse tipo de laboratório tem como objetivo principal imergir os estudantes na cultura e nos aspectos sociais do ambiente clínico, contribuindo para a formação de valores profissionais essenciais para a prática clínica. Essa experiência busca promover segurança, confiança e a redução da ansiedade, permitindo aos estudantes experimentarem novas abordagens e aceitarem erros como parte integrante do seu processo de aprendizagem. Além disso, a participação nessas atividades está associada ao desenvolvimento da autonomia, da competência e da colaboração entre os pares ^( 2 )^.

Diversos elementos influenciam a motivação dos estudantes, incluindo o estilo e a motivação dos docentes, as atividades de ensino, a organização curricular, o contexto acadêmico e a inserção precoce nos ambientes de prática, como recomendado pelas Diretrizes Curriculares Nacionais dos cursos de medicina ^( 16 )^.

Para estimular a motivação dos estudantes no ensino superior, os docentes devem atuar como facilitadores, adotando métodos dinâmicos e criativos que despertem o interesse dos estudantes. O uso de métodos ativos de ensino promove maior motivação para a aprendizagem, uma vez que estudantes motivados buscam compreender e encontrar significado no conhecimento adquirido. Ao abordar a motivação dos estudantes, a ativação de mecanismos de estímulo pode intervir na dinâmica de ensino e de aprendizagem, levando a impactos notáveis no processo de aquisição de conhecimento, bem como na autonomia, no conhecimento e na autoconfiança ^( 7 )^. Um estudo que utilizou métodos ativos demonstrou valores de motivação intrínseca aumentados e regulação externa diminuída ^( 17 )^

Abordando inúmeras situações nas quais o estudante vivencia a aplicabilidade de sua prática profissional, o que pode levá-lo a internalizar a necessidade de aprender para ser um bom profissional, o uso do ensino baseado em simulação é uma ferramenta poderosa por meio da aplicação de treinos de habilidades e cenários simulados ^( 5 )^. Neste estudo, as repercussões do treino de habilidades e dos cenários simulados foram benéficas sobre a motivação, elevando as médias dos escores motivacionais após a realização das atividades dos dois grupos quanto à Regulação Identificada e Motivação Intrínseca, e diminuição da Regulação Externa e Amotivação (Tabela 3 Tabela 2), assim como já encontrado em estudos anteriores ^( 18 )^.

A Motivação Intrínseca está relacionada ao prazer inato pela realização da atividade, sem interesse em ganhos externos ^( 6 )^. A Regulação Identificada aponta que os estudantes internalizaram os motivos pelos quais a atividade estava sendo realizada e, mesmo que não haja interesse inato, reconhecem sua importância para a prática profissional ^( 6 )^.

A Motivação Autônoma, que abrange os itens anteriores, é impulsionada por fatores internos e está alinhada às necessidades, valores e interesses individuais do estudante. Embora possa haver uma fonte externa de motivação, como uma recompensa ou incentivo, quando a motivação autônima predomina, o estudante internaliza os motivos da atividade e reconhece sua relevância para seus objetivos pessoais, valores ou identidade ^( 6 )^.

A Motivação Autônoma é considerada o tipo mais desejável de motivação a ser despertada nos estudantes, pois está associada a melhores resultados de aprendizagem, maior desempenho, empenho e bem-estar ^( 6 )^. No contexto da educação médica, estudantes com motivação autônoma têm maior probabilidade de apresentar comportamentos de aprendizagem proativos, como a busca de experiências clínicas desafiadoras e o envolvimento em práticas reflexivas ^( 17 )^.

O aumento significativo da motivação intrínseca, observado após a participação em cenários clínicos simulados, pode estar relacionado aos significados da autonomia proporcionados por estas atividades. Os cenários clínicos simulados permitem que os estudantes associem as habilidades aprendidas nos treinos à prática de competências, aumentando sentimentos de autoconfiança, autoeficácia e satisfação, especialmente quando acompanhadas por *feedback* e suporte emocional adequados ^( 18 )^.

No que diz respeito ao aumento da Motivação Intrínseca, os cenários simulados comparados ao treino de habilidades (p=0,011) e à diminuição da Amotivação (p=0,035) percebidas no grupo intervenção desta amostra relacionam-se a estudos que indicam que intervalos maiores entre as atividades de ensino favorecem processos de consolidação, como repetições mentais, integração com conhecimentos prévios, fortalecimento de conexões neurais, aumento da familiaridade e da fluência com o tema, autoavaliação e revisão autônoma ^( 19 )^. Esses fatores podem ter implicações na competência, autoeficácia, pertencimento, satisfação e autonomia para o desenvolvimento da atividade proposta. Tais achados vão ao encontro de estudos realizados com estudantes do ensino básico, médio e com graduandos em ciências biológicas, que compararam a motivação para aprendizagem em estudantes submetidos a atividades com desenho instrucional com aplicação de atividades espaçadas, resultando em melhores níveis motivacionais para aprendizagem ^( 20 - 22 )^.

Entretanto, nesta amostra, não foi possível confirmar a hipótese de que o espaçamento entre o treino de habilidades e os cenários simulados aumentariam significativamente a motivação dos estudantes (Teste de Mann-Whitney>0,05). Há inúmeros estudos que apontam os benefícios de intervalos temporais em atividades simuladas, em comparação àquelas realizadas de forma subsequente. No entanto, esses estudos avaliam os efeitos à retenção de competências, habilidades e o conhecimento, sem julgamentos específicos quanto seus efeitos motivação para aprendizagem ^( 5 , 23 )^.

A motivação desempenha um papel crucial no desempenho de estudantes de medicina durante o treinamento de habilidades e nos cenários simulados. Estudantes motivados não apenas se engajam mais nas atividades de simulação, mas também demonstram melhoria do conhecimento e desenvolvimento de atitudes essenciais para a prática clínica. O aprimoramento do ensino em saúde, especialmente por meio da simulação, fortalece a preparação dos futuros médicos, permitindo que cheguem aos estágios mais seguros e capacitados. O potencial de um estudante motivado se estende para além da formação, influenciando positivamente a qualidade da assistência prestada e a segurança do paciente ao longo de toda a carreira profissional. Portanto, é fundamental criar ambientes de aprendizagem que incentivem a motivação e maximizem os benefícios da simulação, garantindo uma formação médica mais eficaz e, consequentemente, um atendimento mais seguro e qualificado aos pacientes ^( 24 )^.

Quanto às limitações do estudo, a amostra foi composta por estudantes de uma única instituição, o que pode limitar a generalização dos resultados para outras populações acadêmicas. Ademais, fatores individuais que influenciam a motivação, como características pessoais e experiências prévias, não foram explorados em profundidade.

Apesar das limitações, os achados contribuem para o aprimoramento do ensino baseado em simulação, oferecendo suporte empírico para o planejamento pedagógico em cursos da área da saúde. A identificação de impactos motivacionais associados ao treino de habilidades e aos cenários simulados reforça a importância de estruturar as atividades de forma progressiva e estrategicamente espaçada.

## Conclusão

Os resultados demonstraram aumento da Motivação Intrínseca (p=0,011) e diminuição da Amotivação (p=0,035) dos estudantes que realizaram o treino de habilidades e os cenários simulados com um maior intervalo de tempo entre suas execuções. Tais resultados são relevantes para o planejamento das atividades acadêmicas, para que sejam motivadoras e remetam a novos propósitos que possam explorar de forma mais apurada os impactos motivacionais em estudantes de medicina. O presente estudo também pode fomentar novas abordagens sobre o potencial da simulação na motivação de estudantes e os diversos desfechos após a experiência simulada, tanto na formação, como ao longo da vida profissional daqueles que desfrutaram do referido método de ensino.

## Data Availability

Todos os dados gerados ou analisados durante este estudo estão incluídos neste artigo publicado.

# Translation: Implicaciones del entrenamiento de habilidades y escenarios simulados en la motivación para el aprendizaje de los estudiantes de medicina: estudio experimental*

**DOI:** 10.1590/1518-8345.7661.4625

**Authors:** Barbara Casarin Henrique-Sanches, Raphael Raniere de Oliveira Costa, Rodrigo Guimarães dos Santos Almeida, Rodrigo Magri Bernardes, Alessandra Mazzo

**Affiliations:** 1 Universidade de São Paulo, Escola de Enfermagem de Ribeirão Preto, Centro Colaborador de la OPS/OMS para el Desarrollo de la Investigación en Enfermería, Ribeirão Preto, SP, Brasil; 2 Becaria de la Coordenação de Aperfeiçoamento de Pessoal de Nível Superior (CAPES), Brasil; 3 Universidade Federal do Rio Grande do Norte, Escola Multicampi de Ciências Médicas, Caicó, RN, Brasil; 4 Universidade Federal do Mato Grosso do Sul, Instituto Integrado de Saúde, Campo Grande, MS, Brasil; 5 Universidade de São Paulo, Faculdade de Medicina de Bauru, Bauru, SP, Brasil; 6 Becaria de la Fundação de Amparo à Pesquisa do Estado de São Paulo (FAPESP), Brasil

**Keywords:** Capacitación de Recursos Humanos en Salud, Educación Médica, Estudiantes de Medicina, Entrenamiento Simulado, Motivación, Aprendizaje

## Abstract

verificar el efecto del entrenamiento de habilidades y de los escenarios simulados realizados de forma subsecuente o tardía en la motivación para el aprendizaje de los estudiantes de medicina.

estudio experimental pre y postest con grupo control e intervención. Participaron 50 estudiantes de segundo año de medicina, aleatorizados en dos grupos: grupo control (exposición dialogada, entrenamiento de habilidades y escenarios simulados después de 12 h) y grupo intervención (exposición dialogada, entrenamiento de habilidades y escenarios simulados después de 21 días). La Escala de Motivación Situacional fue utilizada para el análisis.

todos los estudiantes presentaron un aumento en la motivación. En el grupo control, la motivación intrínseca aumentó antes y después del escenario simulado (p=0,011). En el grupo intervención, la motivación intrínseca aumentó antes y después del entrenamiento de habilidades (p=0,013), antes y después del escenario simulado (p=0,024) y después del entrenamiento de habilidades comparado con el escenario simulado (p=0,011), con una reducción de la motivación (p=0,035).

el entrenamiento de habilidades y los escenarios simulados aumentan la motivación de los estudiantes. Sin embargo, es necesario comprender mejor los impactos de los diferentes intervalos entre las actividades.

## Destacados

**(1)** Las prácticas simuladas aumentan significativamente la motivación intrínseca de los estudiantes. **(2)** Intervalos temporales más largos entre las actividades resultan en mayor motivación. **(3)** La optimización temporal de las actividades puede mejorar el compromiso de los estudiantes.

## Introducción

La educación es un campo amplio que busca mejorar las estrategias de aprendizaje, considerando aspectos neurocognitivos y conductuales, como la cognición, las emociones y la motivación ^( 1 )^. En la enseñanza en salud, la simulación clínica destaca como un enfoque eficaz, permitiendo la integración y aplicación de conocimientos cognitivos, psicomotores y actitudinales ^( 2 )^. Sin embargo, su uso requiere una planificación cuidadosa, con objetivos claros y materiales apropiados ^( 3 )^.

Para que la simulación sea eficaz, las actividades deben organizarse de forma progresiva, permitiendo el desarrollo estructurado de las competencias técnicas y comportamentales de los estudiantes ^( 4 )^. El entrenamiento de habilidades, orientado a la mejora técnica y actitudinal, generalmente ocurre mediante la práctica repetitiva con modelos anatómicos y otros recursos ^( 4 )^. Por otro lado, los escenarios simulados, que buscan reproducir situaciones clínicas complejas en un entorno controlado, promueven el desarrollo del razonamiento clínico, la toma de decisiones y el trabajo en equipo ^( 4 - 5 )^. Para optimizar el aprendizaje, los entrenamientos de habilidades suelen preceder a los escenarios simulados, respetando la progresión de complejidad ^( 5 )^.

La motivación desempeña un papel central en el compromiso y el aprendizaje de los estudiantes, siendo influenciada por factores internos y externos ^( 6 )^. Según la Teoría de la Autodeterminación, la motivación puede ser intrínseca, impulsada por el interés genuino y la satisfacción personal, o extrínseca, orientada por recompensas externas y reconocimiento social ^( 6 )^. La satisfacción de las necesidades psicológicas básicas – autonomía, competencia y pertenencia – fortalece la motivación y facilita la internalización de la motivación extrínseca ^( 6 )^.

Los estudios sobre la motivación en la enseñanza basada en simulación apuntan a ventajas significativas, como un entorno positivo que favorece el desarrollo de habilidades técnicas, interpersonales, autonomía, sentido de pertenencia y autoconfianza ^( 2 , 7 - 8 )^. Sin embargo, aún existen lagunas en el conocimiento sobre cómo diferentes diseños de prácticas simuladas (entrenamiento de habilidades, escenarios simulados e intervalos entre actividades) influyen en la motivación para el aprendizaje.

Por lo tanto, este estudio tiene como objetivo verificar el efecto del entrenamiento de habilidades y los escenarios simulados, realizados de forma subsecuente o tardía, en la motivación para el aprendizaje de los estudiantes de medicina.

## Método

### Diseño del estudio

Se trata de un estudio experimental del tipo pretest y postest, siguiendo las directrices del CONSORT ^( 8 )^ para investigaciones basadas en simulación. El estudio adoptó un diseño paralelo, con asignación 1:1 de los participantes en dos grupos distintos y con cegamiento. La recolección de datos se realizó entre los meses de abril y junio de 2022, y no hubo alteraciones metodológicas después de su inicio ^( 9 )^.

### Lugar del estudio

Este estudio se realizó en el Laboratorio de Habilidades y Simulación de una universidad pública en el interior del estado de São Paulo, Brasil, con estudiantes de un curso de medicina. El mencionado curso tiene su proyecto político-pedagógico basado en métodos activos de aprendizaje, incluyendo la simulación clínica. El laboratorio de habilidades y simulación es un espacio físico y estructurado que contiene materiales y equipos de diversas especificidades. Además, cuenta con recursos humanos que actúan como facilitadores y actores profesionales.

### Población y muestra

La población elegible estuvo compuesta por estudiantes de segundo año de la carrera de medicina, y la muestra fue por conveniencia.

Se incluyeron en el estudio los estudiantes debidamente matriculados en segundo año de la carrera de grado, mayores de 18 años, que completaron todas las actividades y llenaron íntegramente los instrumentos de recolección de datos. Fueron excluidos de la muestra los estudiantes que se ausentaron de cualquier actividad de la investigación o que realizaron el llenado incompleto de los instrumentos de recolección de datos.

Cincuenta y seis estudiantes participaron de la primera etapa (clase expositiva en ambiente virtual de aprendizaje). Para las etapas 2 y 3, los participantes fueron asignados aleatoriamente a dos grupos: Grupo Intervención (n=28), quienes realizaron el entrenamiento de habilidades seguido por un intervalo de 12 horas y la realización de escenarios simulados; y Grupo Control (n=28), quienes realizaron el entrenamiento de habilidades seguido de un intervalo de 21 días para la realización de los escenarios simulados.

Tras la aplicación de los criterios de inclusión, la muestra final estuvo constituida por 50 estudiantes. El proceso de selección, asignación y seguimiento de los grupos se presenta en la Figura 1.

**Figura 1-:** Diagrama de flujo. Adaptado del CONSORT ^( 8 )^

### Desenlace

Los resultados primarios de este estudio preveían la existencia de diferencias estadísticamente significativas en los niveles de motivación para el aprendizaje entre los estudiantes de medicina asignados a los diferentes grupos experimentales, así como variaciones significativas en las puntuaciones de motivación entre los diferentes momentos de evaluación dentro de cada grupo.

### Aleatorización

La asignación de los participantes se realizó mediante una aleatorización simple, utilizando la función RAND() de *Microsoft Excel* ^®^. Cada participante fue registrado según su número de matrícula universitaria, organizado secuencialmente en una hoja de cálculo, de modo que se aplicó la secuencia de aleatorización. La secuencia de asignación fue generada por un investigador independiente, que no tuvo contacto con los participantes durante la intervención y, posteriormente, fue entregada a los facilitadores responsables de la aplicación de las actividades experimentales.

### Cegamiento

Debido al diseño del estudio, no fue posible cegar a los facilitadores, ya que necesitaban dirigir las actividades en los momentos específicos de cada grupo. Sin embargo, los participantes no conocían la variable temporal analizada en el impacto entre el entrenamiento de habilidades y los escenarios simulados, lo que redujo el riesgo de sesgo de expectativa y respuesta. Además, el análisis estadístico fue realizado por un investigador cegado para la asignación de los grupos, minimizando posibles sesgos en la interpretación de los resultados.

### Desarrollo del estudio

Las intervenciones se realizaron durante las actividades académicas a lo largo del módulo de envejecimiento y tuvieron como tema principal “Evaluación, prevención y tratamiento de heridas crónicas”. La actividad estuvo compuesta por clases expositivas, entrenamiento de habilidades y escenarios de simulación.

La fundamentación teórica de la actividad se basó en una revisión bibliográfica y en las recomendaciones de la guía *European Pressure Ulcer Advisory Panel*, *National Pressure Injury Advisory Panel* y de la *Pan Pacific Pressure Injury Alliance* ^( 10 )^, así como en las directrices de la *International Nursing Association for Clinical Simulation and Learning* ^( 4 )^ y en la Teoría del Aprendizaje Experiencial de Kolb ^( 11 )^.

Después de la planificación y construcción, las actividades fueron validadas por expertos respecto a su validez de cara y de contenido y fueron probadas antes de su aplicación. En esta primera etapa, no se sugirieron alteraciones. Las actividades fueron desarrolladas por facilitadores calibrados y se dividieron en tres momentos principales, cuyas configuraciones detalladas, incluidos los objetivos de aprendizaje, los recursos utilizados y la duración, están descritas en la Figura 2.

Etapa 1 – Clases Expositivas Asíncronas: se impartieron dos clases expositivas asincrónicas, cada una con una duración de dos horas, disponibles en un entorno virtual de aprendizaje. Los contenidos abordaron la anatomía de la piel, los efectos del envejecimiento cutáneo, la fisiopatología de las heridas crónicas y las estrategias de prevención y tratamiento, incluidas las coberturas, terapias compresivas y terapia por presión negativa. Además de las clases, los estudiantes tuvieron acceso a materiales complementarios, como videos instructivos y guías clínicas, disponibles durante 30 días en el entorno virtual. Durante este período, también se realizó el llenado del cuestionario de caracterización de los participantes.

Etapa 2 – Entrenamiento de Habilidades: en la segunda etapa, los estudiantes participaron de un entrenamiento práctico supervisado, enfocado en la evaluación de riesgos para lesiones en pacientes diabéticos con neuropatía y en el desbridamiento de necrosis de coagulación. El entrenamiento siguió un guion estructurado, con demostración práctica, ejecución supervisada y retroalimentación formativa al final de la actividad.

Etapa 3 – Escenarios Simulados: se realizaron dos escenarios clínicos simulados, cada uno con 90 minutos de duración, que incluyeron *pre-briefing* (10 min), simulación (20 min) y *debriefing* estructurado con *feedback* formativo (60 min).

Los casos abordaron situaciones de atención en unidades básicas de salud, siendo: Escenario 1 – Paciente diabético tipo 2 con insensibilidad en los miembros inferiores, hiperqueratosis y lesiones fúngicas, simulando una consulta médica en atención primaria.

Escenario 2 – Paciente parapléjico con lesiones por presión en diferentes etapas, necesitando desbridamiento instrumental de tejidos no viables. El escenario se realizó de forma híbrida, utilizando un actor profesional y un simulador de baja fidelidad (pelvis acoplada al paciente simulado).

Durante los *debriefings*, los estudiantes reflexionaron sobre la experiencia, recibiendo feedback estructurado sobre el desempeño clínico, la seguridad en la ejecución de los procedimientos y los aspectos comunicacionales de la consulta.

La Figura 2, a continuación, presenta un desglose de las estrategias de enseñanza, los objetivos de aprendizaje, los recursos utilizados y el tiempo de intervención en cada etapa del estudio.

**Figura 2-:** Estructura de las estrategias de enseñanza, los objetivos de aprendizaje, los recursos utilizados y el tiempo de intervención. Bauru, SP, Brasil, 2024

**Estrategia de enseñanza y aprendizaje**	**Objetivos de aprendizaje**	**Recursos utilizados**	**Tiempo de intervención**
Clases expositivas y asincrónicas disponibles en entorno virtual de aprendizaje	Reconocer la anatomía de la piel; Comprender la acción del proceso de envejecimiento en el tejido cutáneo, así como la etiología y fisiopatología de las lesiones por presión, vasculogénicas y por neuropatía diabética; Identificar y clasificar tejidos viables, no viables y el estadio de las lesiones por presión; Reflexionar sobre las estrategias de prevención de lesiones y el manejo de las lesiones en cuanto a la indicación de coberturas, correlatos, terapias compresivas elásticas, inelásticas y terapia por presión negativa.	Videos de apoyo: evaluación del pie del paciente diabético, medición del índice tobillo-brazo, aplicación de bota de Unna, terapia multicapa, terapia por presión negativa; Materiales de apoyo: *guidelines* y escalas de evaluación de riesgo de desarrollo de lesiones por presión.	4 horas
Entrenamiento de habilidades	Realizar prueba de evaluación de sensibilidad y rastreo en pies de pacientes con riesgo de desarrollar neuropatía diabética. Realizar desbridamiento instrumental de necrosis por coagulación y curativo oclusivo.	Camilla; Diapasón de 128 Hz; Estensiometro Semmes-Weinstein con monofilamento de 10 g Pie de cerdo *in natura* ; Soplete culinario; Guantes estériles; Cuchilla de bisturí; Mango de bisturí; Pinza anatómica; Pinza de ratón; Bandeja para procedimiento; Gasa estéril; Coberturas y correlatos simulados; Cinta microporosa; Tijeras; Filme de poliuretano; Contenedor para material orgánico; Contenedor para material contaminado; Caja rígida para desecho de material punzante.	4 horas
Escenario Simulado 1	Primarios: Vivenciar una atención en una unidad básica de salud con paciente diabético con sensibilidad en los pies comprometida, con presencia de hiperqueratosis y lesiones fúngicas. Secundarios: Desarrollar la comunicación médico-paciente; Adoptar los principios de bioseguridad; Desarrollar la competencia de juicio clínico; Prestar atención de forma segura, basada en evidencia científica; Implementar medidas de prevención y orientación sobre autocuidado para la prevención de lesiones.	Diapasón de 128 Hz; Estensiometro Semmes-Weinstein con monofilamento de 10 g; *Moulage* ; Actor; Algodón; Alcohol 70%.	90 minutos Pre- *Briefing*: 10 min Escenario: 20 min *Debriefing*: 60 min
Escenario Simulado 2	Primario: Vivenciar una atención en una unidad básica de salud con paciente parapléjico con lesiones por presión. Secundarios: Desarrollar la comunicación médico-paciente; Adoptar los principios de bioseguridad; Desarrollar la competencia de juicio clínico; Prestar atención de forma segura, basada en evidencia científica; Realizar desbridamiento instrumental de tejidos no viables; Realizar curativo con coberturas y correlatos con la indicación correcta; Implementar medidas de prevención de lesiones por presión.	Silla de ruedas; Almohadilla de aire con orificio central; Camilla; Sábanas; Simulador de baja fidelidad (pelvis); Masa de moldeo; Sangre artificial; Quemadura artificial; Cáscara de plátano; Maquillaje en crema roja, marrón, morada y amarilla; Cuchilla de bisturí; Mango de bisturí; Guantes de procedimiento; Guantes estériles; Pinza anatómica; Pinza de ratón; Gasa; Alcohol 70%; Coberturas y correlatos simulados; Tijeras; Cinta microporosa.	90 minutos Pre- *Briefing*: 10 min Escenario: 20 min *Debriefing*: 60 min.

### La intervención y la división de los Grupos Control y Experimental

Después de la Etapa 1, es decir, después de asistir a las clases asincrónicas en el entorno virtual de aprendizaje, los estudiantes fueron aleatorizados en el Grupo Control y el Grupo Experimental. A partir de ahí, el Grupo Control realizó el entrenamiento de habilidades (Etapa 2) y, después de un intervalo de 12 horas, participó del escenario simulado (Etapa 3). Por su parte, el Grupo Intervención realizó el entrenamiento de habilidades (Etapa 2) y, después de un intervalo de 21 días, participó del escenario simulado (Etapa 3). Antes y después del entrenamiento de habilidades, así como antes y después de los escenarios simulados, ambos grupos completaron la Escala de Motivación Situacional ^( 12 )^ . 

### Instrumentos de recolección de datos

Se utilizaron dos instrumentos de recolección de datos. El primero fue un cuestionario con preguntas abiertas y cerradas, relacionadas con la caracterización académica y sociodemográfica de los participantes, incluyendo edad, género, apoyos financieros recibidos de la universidad, experiencia previa con el tema de la simulación. Este instrumento se aplicó después del desarrollo de las actividades en el entorno virtual de aprendizaje.

El segundo instrumento fue la Escala de Motivación Situacional ^( 12 )^ , desarrollada por Guay, Vallerand & Blanchard ^( 13 )^ y traducida y validada al portugués por Gamboa, Valadas & Paixão ^( 12 )^ . La versión en portugués demostró validez de constructo y criterio adecuadas, con análisis factorial confirmatorio, sustentando su estructura en cuatro factores, conforme lo previsto por la Teoría de la Autodeterminación ^( 6 )^ . La confiabilidad de la escala se evaluó mediante el alfa de Cronbach, variando entre 0,77 y 0,89, lo que indica una buena consistencia interna ^( 12 )^ . 

El instrumento está destinado a evaluar la motivación situacional en un contexto educativo y está compuesto por 16 ítems, divididos en 4 categorías: Motivación Intrínseca (“Porque pienso que esta actividad es interesante”), Regulación Identificada (“Porque es para mi propio bien”), Regulación Externa (“Porque lo puedo hacer”) y Amotivación (“Puede haber buenas razones para hacer esta actividad, pero no veo ninguna”). Las respuestas se registraron en una escala Likert, variando de 1 (No corresponde en absoluto) a 7 (Correspondencia exacta).

## Aspectos éticos

Este estudio fue aprobado por el Comité de Ética en Investigación bajo el dictamen nº 4.843.772, y se siguieron todas las recomendaciones de la Resolución 466 de 2012 del Consejo Nacional de Salud ^( 14 )^ . 

## Resultados

La edad media de los participantes fue de 21 años, siendo la mayoría del sexo femenino. La mayoría también recibía apoyo financiero de la universidad, realizaba actividades voluntarias en el Laboratorio de Habilidades y Simulación, y ya había tenido contacto previo con pacientes con heridas crónicas. Los datos sociodemográficos y académicos de caracterización de la muestra, por grupo, se presentan en la Tabla 1 . 

**Tabla 1-:** Perfil sociodemográfico de los estudiantes. Bauru, SP, Brasil, 2024

**Caracterización**		**Total**		**Grupo Intervención**		**Grupo Control**	
		Fr*	**%** ^†^	Fr*	**%** ^†^	Fr*	**%** ^†^
Sexo	Femenino	35	70	16	70	19	70
	Masculino	15	30	7	30	8	30
Edad	Mínimo	18	-	18	-	18	-
	Máximo	32	-	32	-	32	-
	Media	21	-	21	-	21	-
	Mediana	21	-	21	-	21	-
Apoyo financiero de la universidad ^‡^	Recibe	27	54	12	52	15	56
	No recibe	23	46	11	48	12	44
Actividades voluntarias en el LHS ^§^ en otro turno	Realiza	50	100	23	100	27	100
	No realiza	0	0	0	0	0	0
Contacto con paciente con herida crónica	Sí	38	72	17	74	21	78
	No	12	28	6	26	6	22
Lugar de contacto con paciente con heridas	UBS ^||^	19	38	8	35	11	41
	Familiares	7	14	3	13	4	15
	Hospital Universitario	6	12	3	13	3	11
	Centro de Especialidad	6	12	3	13	3	11

*Fr = Frecuencia; ^†^ % = Porcentaje; ^‡^ Apoyo financiero otorgado a los estudiantes para contribuir con su permanencia en los cursos superiores y graduación; ^§^ LHS = Laboratorio de Habilidades y Simulación; ^||^ UBS = Unidad Básica de Salud

La Escala de Motivación Situacional ^( 12 )^ mostró una alta confiabilidad en la muestra estudiada (α=0,873) en todos los ítems. La Tabla 2 presenta la estadística descriptiva de los valores del instrumento en relación con la motivación de los dos grupos de estudiantes, medida antes y después de la realización del entrenamiento de habilidades y de los escenarios simulados. 

**Tabla 2-:** Perfil motivacional para el aprendizaje de los estudiantes antes y después del entrenamiento de habilidades y escenario simulado según la Escala de Motivación Situacional ^( 12 )^ . Bauru, SP, Brasil, 2024

**Período**	**Dominio Escala**	**Grupo Control**				**Grupo Intervención**			
		Media	Max*	Min ^†^	Desviación estándar	Media	Max*	Min ^†^	Desviación estándar
Antes Entrenamiento de Habilidades	Regulación Identificada	6,2	7,0	4,3	0,765	6,3	7,0	4,3	0,724
	Motivación Intrínseca	5,4	7,0	3,0	1,215	5,6	7,0	4.0	0,881
	Regulación Externa	5,9	7,0	2,6	1,300	5,5	7,0	2,6	1,468
	Amotivación	1,5	3,2	1,0	0,702	1,2	2,2	1,0	0,336
Después Entrenamiento de Habilidades	Regulación Identificada	6,2	7,0	4,0	1,019	6,4	7,0	3,0	0,981
	Motivación Intrínseca	5,7	7,0	2,0	1,252	6,1	7,0	4,2	0,895
	Regulación Externa	5,8	7,0	2,3	1,409	5,5	7,0	2,0	1,689
	Amotivación	1,5	4,2	1,0	0,930	1,1	2,0	1,0	0,233
Antes Escenario Simulado	Regulación Identificada	6,2	7,0	4,3	0,860	6,2	7,0	4,6	0,729
	Motivación Intrínseca	5,4	7,0	2,5	1,352	5,4	7,0	4,0	0,922
	Regulación Externa	5,8	7,0	3,0	1,141	5,4	7,0	2,3	1,534
	Amotivación	1,3	4,0	1,0	0,651	1,3	3,5	1,0	0,607
Después Escenario Simulado	Regulación Identificada	6,4	7,0	4,0	0,902	6,3	7,0	5,0	0,788
	Motivación Intrínseca	5,7	7,0	2,7	1,191	5,7	7,0	4,2	0,903
	Regulación Externa	5,9	7,0	3.3	1,197	5,4	7,0	1,3	1,598
	Amotivación	1,3	3,5	1,0	0,584	1,3	2,7	1,0	0,505

*Max = Máximo; ^†^ Min = Mínimo

La muestra presentó una distribución anormal (Kolmogorov-Smirnov p > 0,001), por lo tanto, para comparar la motivación de los estudiantes antes y después de las actividades dentro de cada grupo, se utilizó la Prueba de Wilcoxon. Entre los individuos del mismo grupo, los datos que evidenciaron diferencias significativas en el Grupo Control fueron: Motivación Intrínseca antes *versus* después del escenario simulado, y en el Grupo Intervención, las diferencias se dieron en la Motivación Intrínseca antes *versus* después del entrenamiento de habilidades, antes *versus* después del escenario simulado y después del entrenamiento de habilidades *versus* después del escenario simulado, siendo el último momento también significativo en la Amotivación del Grupo Intervención. Los datos están presentados en la Tabla 3 . 

**Tabla 3-:** Comparación dentro del mismo grupo de los dominios de la Escala de Motivación Situacional ^( 12 )^ en la realización de las actividades propuestas en los cuatro momentos distintos. Bauru, SP, Brasil, 2024

**Momento**	**Dominios Comparados**	ρ* **GC** ^†^	ρ* **GI** ^‡^
Antes y después del entrenamiento de habilidad	Motivación Intrínseca *vs* ^§^ Motivación Intrínseca	-	0,013
Antes y después del escenario simulado	Motivación Intrínseca *vs* ^§^ Motivación Intrínseca	0,011	0,024
Después del entrenamiento de habilidades y escenario simulado	Motivación Intrínseca *vs* ^§^ Motivación Intrínseca	-	0,011
Después del entrenamiento de habilidades y escenario simulado	Amotivación *vs* ^§^ Amotivación	-	0,035

*ρ = Nivel de Significancia Prueba de Wilcoxon; ^†^ GC = Grupo Control; ^‡^ GI = Grupo Intervención; ^§^ *vs* = *Versus*

También se realizó la comparación de los dominios de la Escala de Motivación Situacional ^( 12 )^ entre los Grupos Control y Grupo Intervención en todos los momentos evaluados. Los resultados demuestran que no hubo diferencias significativas en la motivación de los estudiantes en ninguno de los períodos analizados (Mann-Whitney p ≥ 0,05). 

## Discusión

Como se presentó en los resultados, la mayoría de los participantes recibían algún tipo de ayuda financiera. En Brasil, las políticas de permanencia estudiantil se implementaron para mitigar desigualdades, buscando democratizar el acceso a la educación superior. Sin embargo, la permanencia del estudiante en la universidad puede ser desafiante debido a las exigencias y condiciones de los cursos, como los de medicina, que requieren dedicación exclusiva, lo que puede resultar en sufrimiento psicológico y evasión. Para enfrentar estos desafíos y fortalecer el sentimiento de pertenencia, las universidades brasileñas ofrecen ayudas socioeconómicas, como vivienda estudiantil, restaurante universitario y ayuda financiera ^( 15 )^ . El sentimiento de pertenencia juega un papel relevante en el éxito académico y el bienestar emocional del estudiante, reflejando su conexión e identificación con el entorno de aprendizaje. Cuando los estudiantes se sienten parte integral de este entorno, demuestran un mayor compromiso en el proceso de adquisición de conocimientos ^( 7 )^ . 

Se observó que la mayoría de los participantes realizaba actividades voluntarias en el Laboratorio de Habilidades y Simulación. Este tipo de laboratorio tiene como objetivo principal sumergir a los estudiantes en la cultura y los aspectos sociales del entorno clínico, contribuyendo a la formación de valores profesionales esenciales para la práctica clínica. Esta experiencia busca promover seguridad, confianza y la reducción de la ansiedad, permitiendo que los estudiantes experimenten nuevos enfoques y acepten los errores como parte integral de su proceso de aprendizaje. Además, la participación en estas actividades está asociada al desarrollo de la autonomía, la competencia y la colaboración entre los compañeros ^( 2 )^ . 

Diversos elementos influyen en la motivación de los estudiantes, incluidos el estilo y la motivación de los docentes, las actividades de enseñanza, la organización curricular, el contexto académico y la inserción temprana en los entornos de práctica, tal como lo recomiendan las Directrices Curriculares Nacionales de los cursos de medicina ^( 16 )^ . 

Para estimular la motivación de los estudiantes en la educación superior, los docentes deben actuar como facilitadores, adoptando métodos dinámicos y creativos que despierten el interés de los estudiantes. El uso de métodos activos de enseñanza promueve una mayor motivación para el aprendizaje, ya que los estudiantes motivados buscan comprender y encontrar significado en el conocimiento adquirido. Al abordar la motivación de los estudiantes, la activación de mecanismos de estímulo puede intervenir en la dinámica de enseñanza y aprendizaje, llevando a impactos notables en el proceso de adquisición de conocimientos, así como en la autonomía, el conocimiento y la autoconfianza ^( 7 )^ . Un estudio que utilizó métodos activos mostró valores aumentados de motivación intrínseca y disminución de la regulación externa ^( 17 )^ . 

Abordando numerosas situaciones en las que el estudiante vivencia la aplicabilidad de su práctica profesional, lo que puede llevarlo a internalizar la necesidad de aprender para ser un buen profesional, el uso de la enseñanza basada en simulación es una herramienta poderosa a través de la aplicación de entrenamientos de habilidades y escenarios simulados ^( 5 )^ . En este estudio, las repercusiones del entrenamiento de habilidades y los escenarios simulados fueron beneficiosas sobre la motivación, elevando las medias de los puntajes motivacionales después de la realización de las actividades de los dos grupos en cuanto a la Regulación Identificada y Motivación Intrínseca, y disminución de la Regulación Externa y la Amotivación ( Tabla 3 Tabla 2), tal como se encontró en estudios anteriores ^( 18 )^ . 

La Motivación Intrínseca está relacionada con el placer innato por realizar la actividad, sin interés en ganancias externas ^( 6 )^ . La Regulación Identificada señala que los estudiantes internalizaron los motivos por los cuales la actividad se estaba realizando y, aunque no haya un interés innato, reconocen su importancia para la práctica profesional ^( 6 )^ . 

La Motivación Autónoma, que abarca los ítems anteriores, está impulsada por factores internos y está alineada con las necesidades, valores e intereses individuales del estudiante. Aunque puede haber una fuente externa de motivación, como una recompensa o incentivo, cuando predomina la motivación autónoma, el estudiante internaliza los motivos de la actividad y reconoce su relevancia para sus objetivos personales, valores o identidad ^( 6 )^ . 

La Motivación Autónoma se considera el tipo más deseable de motivación a despertar en los estudiantes, ya que está asociada con mejores resultados de aprendizaje, mayor rendimiento, dedicación y bienestar ^( 6 )^ . En el contexto de la educación médica, los estudiantes con motivación autónoma tienen mayor probabilidad de presentar comportamientos de aprendizaje proactivos, como la búsqueda de experiencias clínicas desafiantes y la participación en prácticas reflexivas ^( 17 )^ . 

El aumento significativo de la motivación intrínseca, observado después de la participación en escenarios clínicos simulados, puede estar relacionado con los significados de autonomía proporcionados por estas actividades. Los escenarios clínicos simulados permiten que los estudiantes asocien las habilidades aprendidas en los entrenamientos con la práctica de competencias, aumentando los sentimientos de autoconfianza, autoeficacia y satisfacción, especialmente cuando se acompañan de retroalimentación y apoyo emocional adecuados ^( 18 )^ . 

En cuanto al aumento de la Motivación Intrínseca, los escenarios simulados comparados con el entrenamiento de habilidades (p=0,011) y la disminución de la Amotivación (p=0,035) percibidos en el grupo de intervención de esta muestra se relacionan con estudios que indican que intervalos más largos entre las actividades de enseñanza favorecen los procesos de consolidación, como repeticiones mentales, integración con conocimientos previos, fortalecimiento de conexiones neuronales, aumento de familiaridad y fluidez con el tema, autoevaluación y revisión autónoma ^( 19 )^ . Estos factores pueden tener implicaciones en la competencia, autoeficacia, pertenencia, satisfacción y autonomía para el desarrollo de la actividad propuesta. Tales hallazgos coinciden con estudios realizados con estudiantes de educación básica, media y universitarios en ciencias biológicas, que compararon la motivación para el aprendizaje en estudiantes sometidos a actividades con diseño instruccional que aplicaban actividades espaciadas, resultando en mejores niveles motivacionales para el aprendizaje ^( 20 - 22 )^ . 

Sin embargo, en esta muestra, no fue posible confirmar la hipótesis de que el espaciamiento entre el entrenamiento de habilidades y los escenarios simulados aumentaría significativamente la motivación de los estudiantes (Prueba de Mann-Whitney>0,05). Hay numerosos estudios que apuntan a los beneficios de los intervalos temporales en actividades simuladas, en comparación con aquellas realizadas de forma subsecuente. Sin embargo, estos estudios evalúan los efectos sobre la retención de competencias, habilidades y conocimientos, sin juicios específicos sobre sus efectos en la motivación para el aprendizaje ^( 5 , 23 )^ . 

La motivación desempeña un papel crucial en el desempeño de los estudiantes de medicina durante el entrenamiento de habilidades y en los escenarios simulados. Los estudiantes motivados no solo se involucran más en las actividades de simulación, sino que también demuestran una mejora en el conocimiento y el desarrollo de actitudes esenciales para la práctica clínica. El perfeccionamiento de la enseñanza en salud, especialmente a través de la simulación, fortalece la preparación de los futuros médicos, permitiéndoles llegar a las etapas más seguras y capacitados. El potencial de un estudiante motivado se extiende más allá de la formación, influyendo positivamente en la calidad de la asistencia prestada y la seguridad del paciente a lo largo de toda la carrera profesional. Por lo tanto, es fundamental crear entornos de aprendizaje que fomenten la motivación y maximicen los beneficios de la simulación, garantizando una formación médica más eficaz y, en consecuencia, una atención más segura y calificada a los pacientes ^( 24 )^ . 

En cuanto a las limitaciones del estudio, la muestra estuvo compuesta por estudiantes de una sola institución, lo que puede limitar la generalización de los resultados a otras poblaciones académicas. Además, factores individuales que influyen en la motivación, como características personales y experiencias previas, no fueron explorados en profundidad.

A pesar de las limitaciones, los hallazgos contribuyen al perfeccionamiento de la enseñanza basada en simulación, ofreciendo soporte empírico para la planificación pedagógica en cursos del área de la salud. La identificación de impactos motivacionales asociados al entrenamiento de habilidades y los escenarios simulados refuerza la importancia de estructurar las actividades de manera progresiva y estratégicamente espaciada.

## Conclusión

Los resultados demostraron un aumento de la Motivación Intrínseca (p=0,011) y una disminución de la Amotivación (p=0,035) en los estudiantes que realizaron el entrenamiento de habilidades y los escenarios simulados con un mayor intervalo de tiempo entre sus ejecuciones. Tales resultados son relevantes para la planificación de las actividades académicas, para que sean motivadoras y conduzcan a nuevos propósitos que puedan explorar de manera más precisa los impactos motivacionales en los estudiantes de medicina. El presente estudio también puede fomentar nuevos enfoques sobre el potencial de la simulación en la motivación de los estudiantes y los diversos resultados después de la experiencia simulada, tanto en la formación como a lo largo de la vida profesional de aquellos que disfrutaron de este método de enseñanza.

## Data Availability

Todos los datos generados o analizados durante este estudio están incluidos en este artículo publicado.
